# Association of Intact *dupA* (*dupA*1) rather than *dupA*1 cluster with duodenal ulcer in Indian population

**DOI:** 10.1186/s13099-015-0056-2

**Published:** 2015-03-28

**Authors:** Jawed Alam, Prachetash Ghosh, Mou Ganguly, Avijit Sarkar, Ronita De, Asish K Mukhopadhyay

**Affiliations:** Division of Bacteriology, National Institute of Cholera and Enteric Diseases, P 33, CIT Road, Scheme, XM Beliaghata

**Keywords:** *Helicobacter pylori*, Duodenal ulcer, *dupA*, *dupA* cluster, Non-ulcer dyspepsia, Disease association

## Abstract

**Background:**

The duodenal ulcer promoting gene (*dupA)* and *dupA* cluster in *Helicobacter pylori* have been described as a risk factor for duodenal ulcer development in some populations. Polymorphic gene *dupA* can be divided into two groups, intact *dupA1* (long or short type based on the presence or absence of 615-bp extra sequences at the 5′ region) having complete reading frame and other truncated *dupA2* having frame-shift mutation. This study was aimed to elucidate the role of *dupA* of *H. pylori* and their clusters in the disease manifestation of Indian population.

**Methods:**

A total of 170 *H. pylori* strains were screened for the presence of *dupA, dupA* alleles and *dupA* cluster by PCR and sequencing. Pro-inflammatory cytokine (IL-8) with different *dupA* variant *H. pylori* stimulated gastric epithelial cells (AGS cells) was measured by ELISA.

**Results:**

A total of 50 strains (29.4%) were positive for *dupA* among the tested 170 strains. The prevalence of *dupA*1 in duodenal ulcer (DU) and non-ulcer dyspepsia (NUD) populations was found to be 25.5% (25/98) and 11.1% (8/72), respectively and 16.4% (28/170) of the tested strains had *dupA1*, *cagA* and *vacAs1m1* positive. The distribution of long and short type *dupA1* has not been significantly associated with the disease outcome. The *dupA* cluster analysis showed that 10.2% (10/98) and 8.3% (6/72) strains were positive among DU and NUD, respectively. IL-8 production was significantly higher in *dupA*1^+^*, cagA*^+^, *vacA*^+^ (902.5 ± 79.01 pg/mL) than *dupA*2^*+*^*, cagA*^*+*^*, vacA*^*+*^ (536.0 ± 100.4 pg/mL, *P* = 0.008) and *dupA*^−^, *cagA*^+^, *vacA*^+^ (549.7 ± 104.1 pg/mL, *P* = 0.009). Phylogenetic analysis of *dupA* indicated that the Indian *H. pylori* strains clustered with East Asian strains but distinct from Western strains. This is the first known genetic element of Indian *H. pylori* that is genetically closer to the East Asian strains but differed from the Western strains.

**Conclusions:**

The intact *dupA1* was significantly associated with DU than NUD (*P* = 0.029) but the *dupA1* cluster has no role in the disease manifestation at India (*P* = 0.79). Thus, *dupA1* can be considered as a biomarker for DU patients in India.

## Introduction

Infection caused by *H. pylori* is a growing concern as this pathogen is involved in chronic gastritis, peptic ulcer and multi-step carcinogenic processes of gastric cancer. Gastric cancer is the fourth most common cancer worldwide and the second cause of cancer related deaths [1-3]. Epidemiologically, more than 50% of the world population has been infected by this bacterium with persistent inflammation in their stomachs, which lasts for decades unless treated with antibiotics. However, only 15-20% of infected patients develop gastric or duodenal ulcer (DU) and less than 1% develop gastric adenocarcinoma [4]. Several studies demonstrate that about 50-80% Indian populations have been infected with the *H. pylori* [5,6]. It was shown that mere presence of *H. pylori* in the stomach is not associated with any gastric disease. Besides bacterial genetics, host genetic factors, hygiene, microbiome, medication, food habits along with life-style of the individuals are said to enhance the infections caused by *H. pylori*. In this connection, *H. pylori* bear an arsenal of specific virulence factors. Among them, the cytotoxin-associated gene pathogenicity island (*cag*-PAI) and vacuolating associated cytotoxin gene A (*vacA*) are associated with virulence in Western countries. However, the association of *cag*-PAI and *vacA* of *H. pylori* are not established in the Indian population [7]. A novel virulence factor duodenal ulcer promoting gene (*dupA*), located in the plasticity region of the *H. pylori* genome, homologues to *virB*4 gene, which encodes a component protein of the type IV secretion system (T4SS) has been associated with increased risk of DU and protection against gastric cancer (GC) in East Asian and Western countries [8]. However, the role of *dupA* as a virulence marker is still debated [9-17]. It has been reported that *dupA* gene is highly polymorphic as frameshift mutation found along the length of the gene that leads to truncated protein and the rate of frameshift mutation varies geographically around the world [12,18-20]. Thus, mere detection of *dupA* gene by PCR is not adequate to characterize this variable gene. Accordingly, the *dupA* gene has been classified into two groups; i.e., (i) intact *dupA* without frameshift mutation called *dupA1* and (ii) *dupA* with frameshift mutation that leads to stop codon called *dupA2*.

The full genome sequence analysis of strains SNT49, Shi470 and G27 along with the study conducted on the Okinawa population in Japan revealed that the intact *dupA* has two genotypes: short type of 1.8 kb and long type of 2.5 kb due to an additional length of 615 bp in the 5′ region [21]. The latter has been primarily associated with gastric ulcer and gastric cancer in the Okinawa population [21]. A recent study reported that *dupA* and adjacent 6 *vir* genes homologues (*virB8*, *virB9*, *virB10*, *virB11*, *virD2* and *virD4*) in the plasticity region predicting to form a third T4SS (*tfs3a*) [22] termed as “*dupA* cluster” (*dupA* along with six surrounding gene) has been associated with DU in the United States population [23]. However, the functional role of this *dupA* cluster in endorsing the DU formation in the Iraqi population is not very clear [24]. It is well known that the Indian *H. pylori* strains are genetically distinct than East Asian and Western strains [7].

*H. pylori* infection *in vivo* induces the mucosal production of various cytokines *e.g.* interleukin-8 (IL-8), IL-1b, IL-6 and tumor necrosis factor alpha (TNF-α). IL-8, a potent neutrophil-activating chemokine produced by various cell types, including macrophages, epithelial cells, endothelial cells and T cells. Elevated levels of IL-8 have been reported in a number of inflammatory conditions, including inflammatory bowel disease, cystic fibrosis, psoriasis, rheumatoid arthritis, septic shock, and acute meningococcal infections [25]. IL-8 a chemokine, central to the pathogenesis of *H. pylori*-induced tissue injury [26] and previous reports from other research groups showed that *dupA-*positive strains are associated with high-level of IL-8 production [8,19]. Moreover, our recent study showed that intact *cag*-PAI containing *H. pylori* strains were found more frequently in Kolkata than in southern India indicating regional variations in *H. pylori* gene pools [27]. Considering the genetic diversity of *H. pylori* and associated infection, we have undertaken this study to elucidate the role of *dupA* alleles and their cluster in disease manifestation in Indian population.

## Methods

### *H. pylori* strains

A total of 170 *H. pylori* strains archived in the National Institute of Cholera and Enteric Diseases (NICED) Kolkata were used in this study. These strains were isolated from 98 DU patients and 72 NUD subjects of both sexes (aged between 20 and 65 years) with upper gastrointestinal disorder who underwent endoscopy or gastric surgery at the Hospital of the Institute of Post Graduate Medical Education and Research, Kolkata, and St. John’s Medical College Hospital, Bangalore, India during the year 2004–2010. The NICED Ethical committee had approved the study. The patient information or record was kept blind during the experimental procedures and the disease status was decoded during the data analysis. The *H. pylori* strains were stored in brain heart infusion (BHI) broth (Difco Laboratories) with 15% glycerol at −70°C until further use. These strains were revived using BHI agar (Difco) supplemented with 7% sheep blood, 0.4% IsoVitaleX, amphotricin B (8 μg/ml) (Sigma Chemicals Co., St. Louis, MO), trimethoprim (5 μg/ml), vancomycin (6 μg/ml) and nalidixic acid (8 μg/ml) (all from Sigma). Plates were incubated at 37°C in a double gas incubator (Heraeus Instrument, Germany), which maintains an atmosphere of 5% O_2_, 10% CO_2_ and 85% N_2_. The *H. pylori* culture was reconfirmed by positive reactions in urease, catalase and oxidase tests along with the urease PCR.

### PCR Amplification

*H. pylori* genomic DNA isolation and PCR of *dupA*, *cagA* and *vacA* were done using primers and protocols described previously [17]. The six *vir* genes *virB8*, *virB9*, *virB10*, *virB11*, *virD4* and *virD2* surrounding the *dupA* and forming the *dupA* cluster were amplified with primers described elsewhere [23]. Gene specific primers were used for the amplification of *dupA* genotypes under different PCR condition listed in Table 1. Long type and short type *dupA1* was amplified with primer pair (*SNT49F*/*dupA2499R*) and (*dupAF/dupA2499R*), respectively. Primer *SNT49F* was designed from the start codon of *dupA* of full genome sequenced Indian strain SNT49, located 615 bp upstream to the earlier proposed *dupA* of C142 strain (AB 196363). Primer *dupA2499R* was located on the stop codon (end) of *dupA* of SNT49 strain and 5′ end of the *jhp0919* region of J99 strain. Primer *dupAF* was designed from the start codon of *jhp0917* of strain J99. The PCR conditions were standardized to a final volume of 20 μl containing template DNA (2–20 ng), 2 μl of 10x Buffer (Genei, India), 2.5 mM dNTPs (Genei, India) and 10 pmol of corresponding primers in the presence of 1U of Taq DNA Polymerase (Genei, India) [Table 1].

**Table 1:** **List of primer used for Amplification and sequencing of** ***dupA*** **gene**

**Primer**	**Sequence (5′-3′)**	**PCR cycling condition**	**Amplicon size (bp)**
*SNT49F*	ATGTTTCTTGGTTTAGAGGG	94°C 30 sec, 55°C 30 sec and 72°C 2.5 mins	2499
*dupA2499R*	TCACACATATTGAACATTCTCG		
*dupAF*	ATGAGTTCTGTATTAACAGACTTTG	94°C 30 sec, 50°C 30 sec and 72°C 2 mins	1884
*dupA2499R*	TTAAATACTCTTCCTTATAAGTTTC		
*SNT49F*	ATGTTTCTTGGTTTAGAGGG	94°C 30 sec, 55°C 30 sec and 72°C 1 mins	685
*SNT49R685*	CAGCGTATAAATTCAATAGATC		
*dupAF*	ATGAGTTCTGTATTAACAGACTTTG	94°C 30 sec, 50°C 30 sec and 72°C 1.5 mins	1172
*dupA20R*	CCTAAATTTTTGGCAATTTCTAATAAG		
*dupA16F*	ACAATACTGCTAATACAGATG	94°C 30 sec, 55°C 30 sec and 72°C 1 min	947
*dupA2499R*	TCACACATATTGAACATTCTCG		
*SNT49_470F*	ATGATTTTAAATTATGTAGAGACC	94°C 30 sec, 55°C 30 sec and 72°C 40 secs	623
*SNT49R350*	GCATTAACAATTTTTTTAGCG		
*918 F*	CCTATATCGCTAACGCGCTC	94°C 30 sec, 55°C 30 sec and 72°C 40 secs	791
*jhp0919R*	CTTTTTGTGATTTCATGAAACTC		

### DNA sequencing and phylogenetic analysis

Different fragments of *dupA* were amplified with different set of primers listed in Table 1. Primer walking method was used for the sequencing of full length of *dupA* gene. We used primer pair *SNT49_470F* and *SNT49R350* for confirmation of start codon of long type *dupA* gene and *918 F* and *jhp0919R* for the confirmation of stop codon of *dupA* gene. Primer *jhp0919R* was located from 326 bp to 350 of ORF *jhp0919* of strain J99. The amplified products were purified using the QIAquick PCR Purification Kit (QIAGEN). The purified PCR product was quantified on gel. The intensity of the band compared with *λ hind* III digest. The PCR purified products were sequenced with a Big Dye Terminator v3.1 cycle sequencing kit on an ABI PRISM 3100 genetic Analyzer (Applied Biosystem, USA). The sequences obtained in this study were deposited in GenBank under accession numbers KC894688-KC894692. We performed BLAST to get the identical sequence available on the NCBI. All the sequences obtained were multiple aligned with the known *dupA* sequence from different geographic areas using Clustal W of MEGA6 software (version 6.0.5, AZ, USA). This Maximum Likelihood tree was generated using Tamura 3 parameter model.

### Cell culture and *H. pylori* infection

To measure *in vitro* IL-8 secretion from gastric epithelial cells, AGS **(**human gastric adenocarcinoma cell line) were cultured in RPMI 1640 (HiMedia, Mumbai, India) medium supplemented with 10% fetal bovine serum (Invitrogen, UK) for 3 days at 37°C, under 5% CO_2_. The cells were trypsinized (Gibco BRL), microscopically enumerated, and distributed in a 24-well microtitre plate at a final concentration of 5 X 10^5^ cells/ml (1 ml/well) and incubated for 24 h at 37°C prior to infection. *H. pylori* (multiplicity of infection [MOI] of 100) was added to cultured cells for 8 hours and IL-8 levels in the supernatant was measured in duplicates using a commercially available ELISA kit (Amersham Biosciences Biotrak™ System) following the manufacturer’s instructions.

### Statistical analysis

A univariate analysis was performed to determine the risk of *dupA* alleles and *dupA* cluster in relation to clinical outcome. For univariate analysis, *χ*^2^ test was used. A Probability levels (*P*) value of ≤ 0.05 was considered statistically significant.

## Results

### Distribution of *dupA* alleles

**Figure 1:**
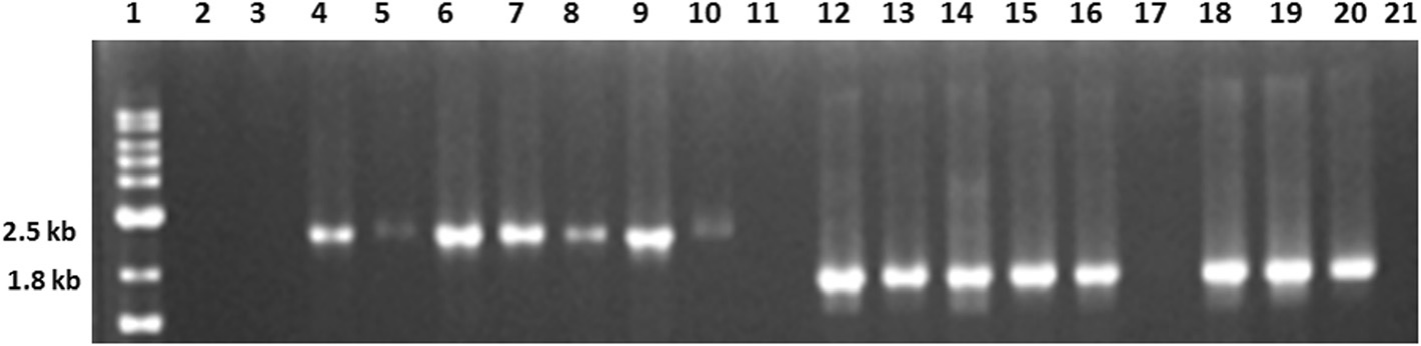
**PCR results of** ***dupA*** **1** ***(*** **long type and short type) amplified with** ***snt49F/2499R*** **and** ***dupAF/2499R*** **sets of primers in representative** ***H. pylori*** **strains.** Lane 1 denoted 1 kb marker (NEB). Lane 2–11 showed the long type *dupA*1 of 2499 bp and lane 12–21 yielded the short type *dupA*1 of 1884 bp, lane 2, 3 and 17 did not produce any amplicon. Lanes 11 and 21 were taken as negative controls.

A total of 170 *H. pylori* strains were isolated from the following two groups: (i) 98 DU patients and (ii) 72 NUD. PCR and sequencing were performed to screen the presence of *dupA* gene with published primers [17] and found that 29.4% (50/170) strains were positive for *dupA*. Frameshift mutations were screened in these *dupA* positive strains by sequencing with primers described in Table 1. It was found that 19.4% (33/170) strains had intact gene without frameshift mutation and hence were considered as *dupA1*. In 10% (17/170) of the strains, frameshift mutation has been detected leading to a premature stop codon and hence the *dupA2* in these strains were truncated. The *dupA1* alleles namely, long type and short type *dupA1* with 2499 bp and 1884 bp amplicons [Figure 1] were detected in 12.3% (21/170) and 7% (12/170) strains, respectively [Table 2]. This data showed that the distribution of long type *dupA1* was more than the short type *dupA1* in the Indian population.

**Table 2:** **Prevalence of** ***dupA (dupA*** **1and** ***dupA*** **2** ***), dupA1*** **alleles and** ***dupA1*** **cluster in Indian population**

	**Total**	**DU**	**NUD**
Number	170	98	72
*dupA*	50/170 (29.4%)	38/98 (38.7%)	12/72 (16.6%)
*dupA1*	33/170 (19.4%)	25/98 (25.5%)	8/72 (11.1%)
long type *dupA*1	21/170 (12.3%)	15/98 (15.3%)	6/72 (8.3%)
short type *dupA*1	12/170 (7%)	10/98 (10.2%)	2/72(2.7%)
*dupA*1 with cluster	16/170 (9.4%)	10/98 (10.2%)	6/72 (8.3%)
*dupA*2	17/170 (10%)	13/98 (13.2%)	4/72 (5.5%)
*cagA*	142/170 (83.5%)	86/98 (87.7)	56/72 (77.7%)
*vacAs1m1*	118/170 (69.4)	71/98 (72.4%)	47/72 (65.2%)
*dupA*1*, cagA, vacAs1m1*	28/170 (16.4%)	22/98 (22.4%)	6/72 (8.3%)

### Prevalence of *vir* genes homologues and *cagA*, *vacA*

All the 6 *vir* genes in 170 strains were tested by primer described by Jung *et al*. [23] and their distribution showed 44.1% of *virB8* (75/170), 30% of *virB9* (51/170), 24.1% of *virB10* (41/170), 27% of *virB11* (46/170), 64.1% of *virD2* (109/170) and 42.9% of *virD4* (73/170) [Table 3]. Twenty strains (11.7%) had *dupA* and all 6 *vir* genes homologues indicating positive for *dupA* cluster and 35 strains had no *vir* gene homologues. A total of 115 strains were positive for various combinations of *vir* gene homologues while lacking some *vir* genes. Further analysis showed that *dupA1* cluster (intact *dupA* gene with 6 *vir* homologues) and *dupA2* cluster (truncated *dupA* with 6 *vir* homologues) was found in 9.4% (16/170) and 2% (4/170) strains, respectively. The *cagA* gene was present in 83.5% (142/170) of the tested strains and 91% (30/33) of the *dupA*1 positive strains from this region. The *vacA* s*1m1* was present in 69.4% (118/170) of the total tested strains and 85% (28/33) of the *dupA1* positive strains [Table 2]. The other two alleles of *vacA*, *s1m2* and *s2m2,* were present in 17.6% (30/170) and 12.9% (22/140) of the strains, respectively (data not shown).

**Table 3:** **Distribution of all six** ***vir*** **gene in Indian population**

**Gene**	**Total (n = 170)**	**DU (n-98)**	**NUD (n = 72)**
*virB8*	75/170 (44.1%)	50/98 (51%)	25/72 (34.7%)
*virB9*	51/170 (30%)	32/98 (32.6%)	19/72 (26.3%)
*virB10*	41/170 (24.1%)	27/98 (27.5%)	14/72 (19.4%)
*virB11*	46/170 (27%)	28/98 (28.5%)	18/72 (25%)
*virD2*	109/170 (64.1%)	65/98 (66.3%)	44/72 (61.1%)
*virD4*	73/170 (42.9%)	42/98 (42.8%)	31/72 (43%)
All six *vir* genes with *dupA (dupA1* and *dupA2)*	20/170 (11.7%)	12/98 (12.2%)	8/72 (11.1%)

### Association of *dupA* genotypes and *dupA* cluster with disease outcome

In this study, 38.7% (38/98) DU and 16.6% (12/72) NUD strains were positive for *dupA* gene by PCR and sequencing of intergenic region of *jhp0917- jhp0918* ORF. Sequence analysis indicated that 66% (33/50) of the *dupA* positive strains had intact *dupA* gene without any frameshift mutation (*dupA1*) and 34% (17/50) strains had insertion or deletion of adenine at different positions leading to a premature stop codon (*dupA2*). Interestingly, the intact *dupA1* was found significantly higher in DU patients (25/98, 25.5%) than in NUD patients (8/72, 11.1%) (*P* =0.02, odds ratio = 2.73, 95% confidence interval = 1.15-6.50). Additional analysis on the type of *dupA1* alleles of *H. pylori* present in the Indian population showed the presence of both long and short types *dupA1* were more in DU patients (15/98, 10/98) than NUD subjects (6/72, 2/72). However, the difference did not reach up to the significant level (*P* = 0.23, odds ratio = 1.98, 95% confidence interval = 0.731-5.40, *P* = 0.073, odds ratio = 3.9, 95% confidence interval = 0.84-18.74). The 6 *vir* genes cluster was detected in 10.2% (10/98) and 8.3% (6/72) of the strains from DU and NUD subjects, respectively with intact *dupA*. This result indicated that there was no significant association of intact *dupA1* cluster with DU (*P* =0.79, odds ratio = 1.25, 95% confidence interval = 0.43-3.61) [Table 2].

### Intact *dupA* and IL-8 production in gastric cancer cells

**Figure 2:**
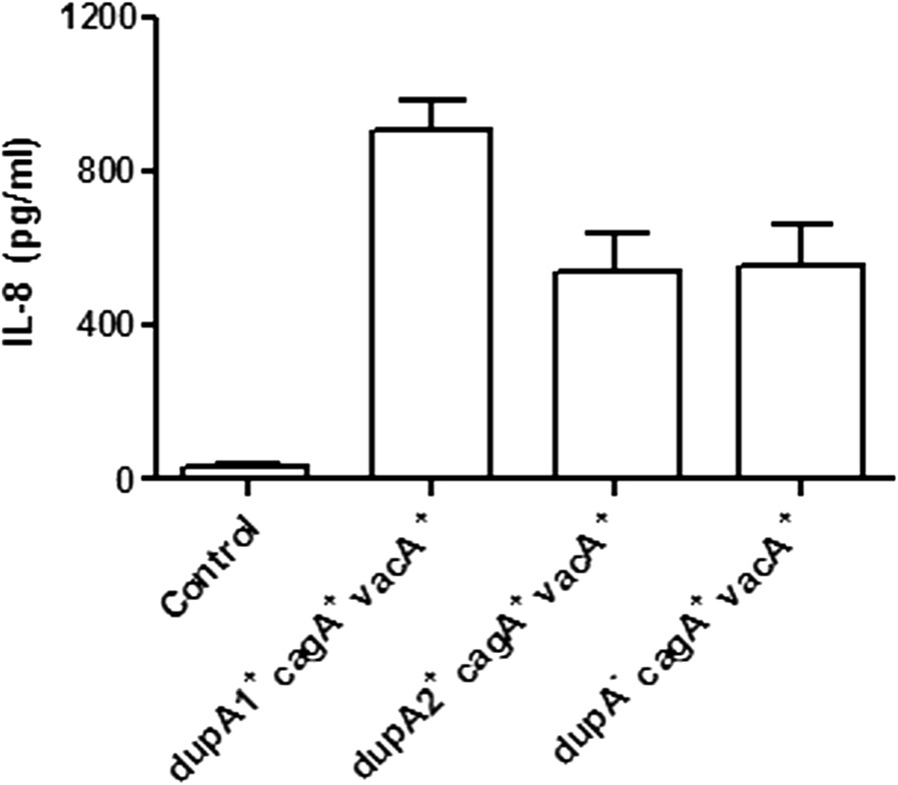
**In vitro IL-8 production from AGS cells co-cultured with 6 (** ***dupA*** **1** ^***+***^ **,** ***cagA*** ^**+**^ **,** ***vacA*** ^**+**^ **) strains, 6 (** ***dupA*** **2** ^**+**^ **,** ***cagA*** ^**+**^ **,** ***vacA*** ^**+**^ **) and 6 (** ***dupA*** ^**−**^ **,** ***cagA*** ^**+**^ **,** ***vacA*** ^**+**^ **) strains.** IL-8 production was significantly higher in *dupA*1^*+*^ (*cagA* ^+^, *vacA* ^+^) compared to *dupA*2^+^ (*cagA* ^+^, *vacA* ^+^) (536.0 ± 100.4 pg/mL, *P* = 0.008) and *dupA*-negative strains (*cagA* ^+^, *vacA* ^*+*^ *)* (549.7 ± 104.1 pg/mL, *P* = 0.009). MOI of *H. pylori* strain was 100 for 8 hours. Experiments were repeated 3 times for the 18 strains. IL-8 in the supernatant was assayed by an ELISA in duplicate. Data are expressed as mean ± standard error.

In general, there was no difference in IL-8 production between *dupA*-positive but truncated (*dupA*2) and *dupA*-negative strains when tested in AGS cells co-cultured with *H. pylori*. However, IL-8 production was significantly higher (902.5 ± 79.01 pg/mL) in the strains with an intact *dupA1* compared with a truncated *dupA2* (536.0 ± 100.4 pg/mL, *P* = 0.008) or with *dupA*-negative strains (549.7 ± 104.1 pg/mL, *P* = 0.009) [Figure 2]. All the strains taken for IL-8 assay were positive for *cagA* and *vacA* which helped us to focus specifically the role of different *dupA* groups*.*

### Phylogenetic analysis of *dupA* gene

**Figure 3:**
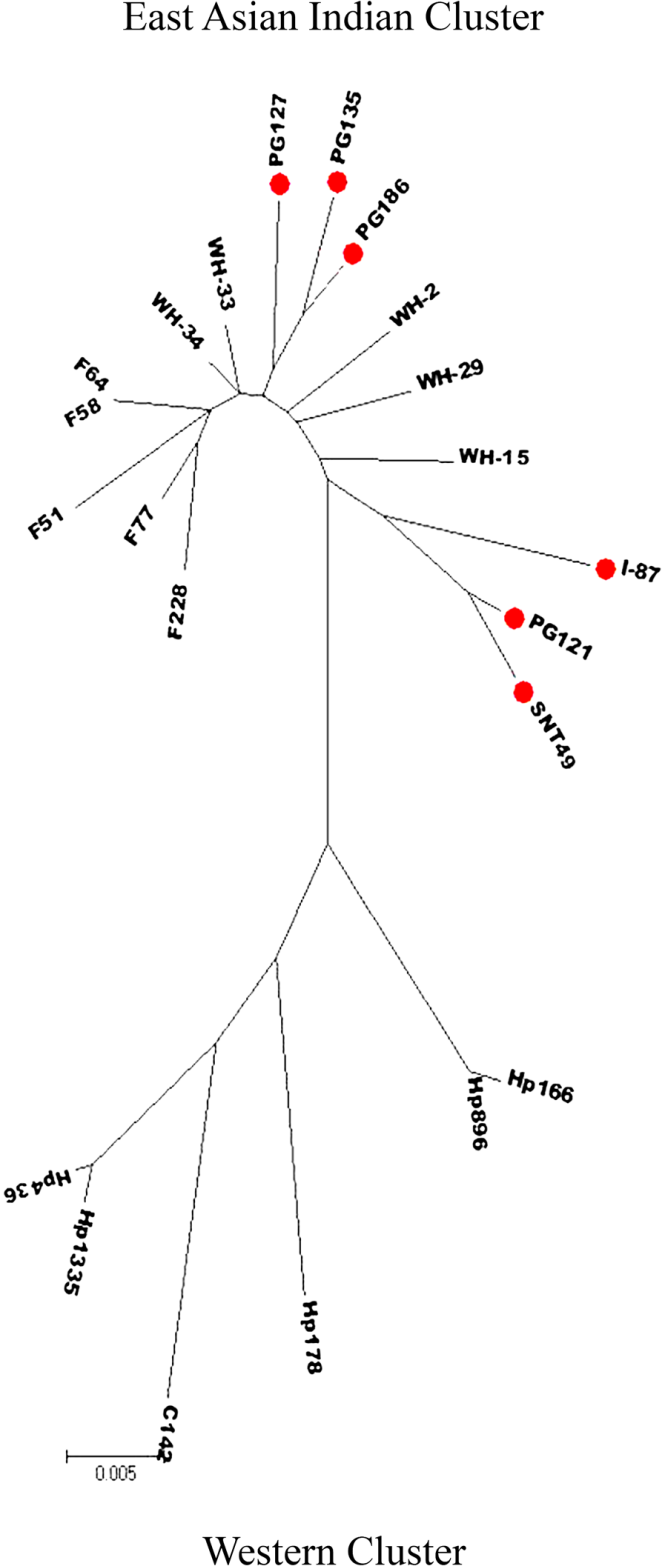
**Phylogenetic tree constructed based on 1.8 kb segment of** ***dupA*** **gene of** ***H. pylori*** **(determined in this study and reported by others).** This Maximum Likelihood tree was generated using Tamura 3 parameter model in MEGA6 software (version 6.0.5, AZ, USA). Representative strains from India are marked in Red dots (●, red circle). Sequences of non Indian strains were used here from public database. Indian and East Asian strains formed one cluster called group I and Brazilian and Colombian strains formed another cluster called group II. The length of the vertical bar indicates the number of nucleotide substitution per site. Bootstrap values of ≥ 70 are indicated at the nodes. *H. pylori* strain designations indicate the geographic origins, as follows: F, Japan; WH, China; PG or I or SNT, India; HP, Brazil; C, Colombia. GenBank: accession no. for the strains used in the study are given in the parentheses: F228 [GenBank: AB617836.1], F77 [GenBank: AB617834.1], F58 [GenBank: AB617835.1], F64 [GenBank: AB617833.1], WH-34 [GenBank: KC707844.1], WH-33 [GenBank: KC707843.1], F51 [GenBank: AB617832.1], PG127 [GenBank: Submission in process], PG135-1a [GenBank: JN379048.1], PG186 [GenBank: KC894690.1], WH-2 [GenBank: KC707837.1], WH-29 [GenBank: KC707842.1], WH-15 ([GenBank: KC707839.1], I-87 [GenBank: KC894692.1], I-121 [GenBank: KC894689.1], SNT49 GenBank: CP002983.1], Hp166.03 [GenBank: HM770857.1], Hp896.95 [GenBank: HM770862.1], Hp178.02 [GenBank: HQ228198.1], C142 [GenBank: AB196363.1], Hp1335.95 [GenBank: HQ228195.1], Hp436.95 [GenBank: HQ228197.1].

Sequence heterogeneity of *dupA* gene was analysed with 22 randomly selected strains from India and other countries (assembled using GenBank data). Phylogenetic analysis of *dupA* sequence revealed the existence of 2 distinct clusters [Figure 3]. The first cluster designated as “Group I” included 5 strains from Japan, 5 from China and 6 from different parts of India. The second group included 6 strains (5 from Brazil and one each from Colombia) and was designated as “Group II”, which was called as the European cluster. Most strikingly, phylogenetic analysis of *dupA* sequence showed that the Indian strains clustered more closely with the East Asian strains.

## Discussion

A novel virulence factor encoded by the duodenal ulcer promoting (*dupA*) gene (homologous to *virB4* and a component of the T4SS) has been found to be associated with DU and increased expression of IL-8 [8]. However, the role of *dupA* as a virulence marker is still a debated issue [9-11,17,19,28,29]. The frameshift mutations in the *dupA* gene with a premature stop codon may have a considerable influence on the protein expression or function of DupA. [12,18-21]. In our study, we have found the rate of frameshift mutations along the length of *dupA* gene was [34% (17/50)] high in the Indian population. Similar trend has also been observed in other populations [19,20,24]. On the other hand, 66% (33/50) of the *dupA* positive strains had no stop codon and were considered as intact *dupA* known as *dupA1*. In consistent with the findings of the other studies, the intact *dupA* (*dupA1*) without frameshift mutation was significantly associated with DU in the Indian population [20,24]. This result reflects that the detection of *dupA* by PCR is not adequate to identify an intact *dupA* because frameshift mutation is common along the length of gene. Hence, *dupA* PCR along with sequencing is mandatory for the detection of intact *dupA* gene. Moura *et al*. [20] reported that intact *dupA* was independently associated with DU and can be used as a disease marker in the Brazilian population. Findings from another study [22] showed that intact *dupA* was more frequent in DU than gastritis but the data did not reach up to the significant level. Nevertheless the intact *dupA* was negatively associated with gastric carcinoma as previously observed by Lu *et al*. [8] and Zhang *et al*. [13]. Takahashi *et al*. [21] reported that there was an additional 615-bp in the 5′ region of *dupA* gene in some Okinawa strains, which classified the *dupA* gene into two alleles: long type (2.5 kb) and short type (1.8 kb) *dupA* gene [21]. The long type *dupA* was significantly associated with gastroduodenal diseases as compared to short type *dupA* [21]. However, this trend has not been detected in the *H. pylori* strains from the Indian population.

Jung *et al*. [23] reported that six additional *vir* genes homologues (*virB8, virB9, virB10, virB11, virD2* and *virD4*) present around the *dupA* gene forming the *dupA* cluster was associated with duodenal ulcer and might play a pathogenic role like other T4SS cluster, similar to *cag* PAI [23]. The present study on additional six genes was carried out to understand the pathogenic associations of *dupA* cluster in Indian strains as they are geographically distinct from Western strains. Results showed that the prevalence of intact *dupA1* with six *vir* genes was not associated with disease outcome in Indian population. The *vir* genes were randomly distributed among DU and NUD patients. In our population, intact *dupA1* (long type or short type) with *vir* genes cluster was not important in promoting DU formation, which is not comparable to the one reported by Jung *et al.* [23] where the intact *dupA* cluster was associated with DU**.** This trend can be linked to our earlier finding in which we have showed that the Indian *H. pylori* strains are genetically distinct from Western and East Asian strains and the intact *cag-*PAI from Kolkata has not associated with disease outcome [7,24]. One study showed that none of the strains from Iraq had all six *vir* gene homologues, but the gene *dupA1* was found to be significantly associated with DU [28]. Considering the importance of *dupA1* cluster in relation to the disease manifestation, there is a need for comprehensive studies around the world. Additionally, intact *dupA* without frameshift mutation should be detected with DupA protein using immunoblot techniques. The intact long type as well as short type *dupA1* gene might produce a functional DupA protein. The primary sequence analysis of *dupA* gene showed that the dupA protein was involved in cell division and peptidoglycan synthesis or modification and was implicated in intercellular chromosomal DNA transfer encodes homologues of VirB4 ATPase, as *jhp0917* region (position 3–201 of *dupA*) contains CagE_TrbE_VirB domain and FtsK/SpoIIIE family. The region from the 3′ region *jhp0917* to *jhp0918* region (position 203–610) is homologue to TraG/TraD family. The FtsK/SpoIIIE domain contains a putative ATP-binding P-loop motif [30,31]. The phylogenetic analysis of *dupA* gene of Indian *H. pylori* strains showed a different pattern as compared to the distribution of other potential virulence genes such as *cagA* and *vacA. cagA* sequence of Indian *H. pylori* strains intermingled with the Western strains but distinct from the east Asian strains. On the other hand, the *vacA* mid region sequence of Indian strains formed a separate cluster from both the Western and the east Asian strains [7]. However, the *dupA* gene from the Indian *H. pylori* strains showed phylogenetic similarity with the East Asian strains and distinct from the Western strains. This is the first known genetic element of Indian *H. pylori* which intermingled with the East Asian strains but differed with the European strains. The exact reason for this *dupA* cluster difference is not known. This *dupA* gene is located in the hypervariable plasticity region and hence there is a possibility that *dupA* gene in particular or whole plasticity region of Indian *H. pylori* might have been acquired from East Asian strains. However, there is a need for independent studies to elucidate the dynamics of *dupA* in different populations around the world. Additionally, it was found that the IL-8 production was significantly associated with DU in intact *dupA1* rather than truncated *dupA2* or *dupA* negative strains. This finding is in accordance with the observation of Hussein *et al*. [24] in the Iraqi population, which showed that IL-8 production was significantly higher in intact *dupA1* strain than truncated *dupA2* or *dupA* negative strains. Our study showed that *H. pylori* strains containing *cagA* and *vacA* can induce IL-8 in cell line assay. We have also demonstrated that *H. pylori* strains containing *cagA, vacA* and *dupA1* can induce IL-8 significantly higher than the strains containing only *cagA* and *vacA*. This result indicates that *dupA1* is an important virulent marker of *H. pylori* in Indian population. Six *dupA2* and 6 *dupA* negative strains included in this study were positive for *cagA* and *vacA* s1m1 genotypes. The presence of *cagA* and *vacA* in these *dupA2* and *dupA* negative strains are capable to induce the IL-8 secretion up to a certain level. Moreover, the prevalence of the six *vir* genes of *dupA* cluster were almost similar in both DU and NUD groups among Indian population. This data suggests that *dupA1* cluster has no role in disease manifestation in the Indian population but *dupA*1 might be considered as an important virulent factor of *H. pylori* for causing duodenal ulcer in Indian population.

Another recent study demonstrated that patients infected with *dupA*-positive *H. pylori* strains had significantly elevated gastric acid output than the *dupA*-negative strains [32]. In addition, Abadi *et al.* [16] detected higher acid resistance of *dupA*-positive strains making them to adopt well under high acidic condition in the stomach milieu. This increased gastric acid output is thought to be typical for an antrum-predominant *H. pylori* infection with an increased risk for DU and it also reduces the risk for the genesis of GUs and GC. Together, these results may explain the associations between the *dupA* gene and an increased risk for DU formation. Several studies conducted in different countries have shown the variable nature of *dupA* with respect to the clinical outcomes. Our findings emphasize the need of examining the frame-shift-mutations or *dupA1* gene polymorphisms in parallel with the disease outcome. In conclusion, the presence of intact *dupA* seems to be important in DU development rather than complete *dupA* cluster (*dupA* with six *vir* genes). Thus, *dupA1* may possibly act as a potential biomarker for disease manifestation in the Indian population.
